# Society News

**Published:** 1914-09

**Authors:** 


### Society News.

THE Navarro County Medical Society held its regular monthly meeting August 3d. Dr. Davis and Dr. Pierce, of Dallas, were visitors at the meeting.

The Falls County Medical Society met in Marlin August 3d. The following program was carried out:

"Some Surgical Difficulties Made Clear by the Use of the X-ray," Dr. R. T. Wilson.

"Some Harmful Effects of Intestinal Toxins," Dr. J. J. Terrell.

After the program was finished the doctors were entertained in the home of Dr. Rice.

The Milam County Medical Society held a very interesting meeting on August 11th at Cameron, Texas. There was a large attendance, and every doctor present declared that the meeting was most interesting and helpful. The next meeting will be held at Rockdale on October 6th.

On August 12th the Nacogdoches County Medical Society met in regular session, and carried out a very important program. Among other things, it was decided to have the meeting at a different place each month. Appleby was chosen as the next place of meeting, and Monday, September 14th, was the time for the meeting.

The Panhandle District Medical Society held its mid-summer meeting in Amarillo on June 26th and 27th. The meeting was largely attended and thoroughly enjoyed by those present. The visiting doctors were given a luncheon and a smoker in the evening. The next meeting will be held in January at Memphis, Texas.

The Hood-Somervell County Medical Society met in Granbury August 5th. Dr. A. R. Jarrett was elected chairman pro tem. The minutes of the last meeting were read. Members present were: Drs. A. R. Jarrett, G. N. Lancaster, E. L. Menefee, E. H. Morgan and H. L. Wilder.

Dr. G. N. Lancaster reported a clincial case of a child two years old that was bitten by some insect that caused hematuria, jaundice fever, edema of the affected side and dilated pupils. The paper was discussed by all present.

Dr. Jarrett reported a case of a man who had been jaundiced for about a year. Tumor and pain in right anterior lumbar region. Autopsy showed tumor obstructing stomach, gall ducts, etc., that proved to be a carcinoma. Discussed by all.

Dr. Wilder read a paper on "Infant Feeding." Discussed by Drs. Lancaster and Jarrett.

The program committee for the picnic reported.

Motion prevailed to receive and discharge the committee.

Dr. E. H. Morgan appointed program committee for the next meeting in Granbury.

Program for the next meeting in Glen Rose September 2d is as follows:

H. L. Wilder, "Blood Pressure as an Aid in Diagnosis."

W. H. Powell, "Diagnosis of Typhoid."

G. N. Lancaster, "Causes of Hematuria."

E. L. Menefee, "Diagnosis of Scarlet Fever."

The La Salle-Frio County Medical Society meets quarterly. The next meeting will be held Tuesday September 15th. The program is as follows:

Dr. C. S. Venable, "Surgery."

Dr. B. T. Young, "Internal Medicine."

Dr. B. E. Pickett, "Acute Ileocolitis."

Dr. Jesse W. Hale, "Ectopic Pregnancy."

There will be some kind of entertainment for visitors after the regular meeting. Our last meeting was held June 16th with a fine program, and was well attended.

Members present: Jesse W. Hale, Fowlerton; Elmer Howard and M. A. Wickware, Pearsall; E. F. Gates, Dilley; H. T. Wichmann and Robt. L. Graham, Cotulla.

Visitors: Drs. Cunningham, Burleson and Lowery, of San Antonio.

After the regular meeting they went to the river for a fish fry, where we were abundantly fed with fish and black coffee by the friends of Drs. Howard and Wickware, who were at that time camping on the river.

We are not at all selfish and are glad to have visitors, and extend the invitation to the Austin doctors and others who would like to have an outing and the benefit of a good medical society.

All we ask is to let us know if you will attend.

ROBT. L. GRAHAM,
Secretary.

The Scurry-Dickens-Kent County Medical Society met at the courthouse in Snyder, Texas, Tuesday, September 1st, at 7:30 p. m. The following program was carried out:

"Diagnosis and Treatment of Acute Gastro-Intestinal Disturbances in Children," Dr. J. M. Bannister, Snyder, Texas.

"Diagnosis and Treatment of Typhoid Fever," Dr. J. T. Whitmore, Snyder, Texas.

L. E. TRIGG,
Secretary.

